# Effort angina in a patient with advanced coronary artery disease. Role played by coronary angiography, Ivus and cardiac CT: case report

**DOI:** 10.1186/1476-7120-6-48

**Published:** 2008-09-24

**Authors:** Domenico M Zardi, Enrico M Zardi, Andrea Berni, Cristiana Nannini, Biagio Andrea Pace, Stefano Santucci, Massimo Volpe

**Affiliations:** 1 Division of Cardiology, II Faculty of Medicine, University of Rome "La Sapienza", Sant'Andrea Hospital, Rome, Italy; 2 Department of Clinical Medicine, "*Campus Bio-Medico*" University, Rome, Italy

## Abstract

Coronary angiography is considered to be the gold standard technique for assessing the severity of obstructive luminal narrowing; however, in a few circumstances it may be misleading. In these cases, cardiac computed tomography (CT) and intravascular ultrasound (IVUS) may help to give a correct interpretation.

In this report, we describe the case of a 62-year-old man whose effort angina was first evaluated with coronary angiography, but whose severe stenosis of the right coronary artery was only observed on cardiac CT and IVUS. This additional diagnosis promptly resulted in a therapeutic approach with percutaneous transluminal coronary angioplasty (PTCA).

## Background

Advanced coronary artery disease (CAD) may be caused by multiple stenotic lesions in which plaque remodelling and thrombus play an important role. Since the 60's, coronary angiography has been proven to be the gold standard invasive method for assessing CAD and providing information about the silhouette of the coronary artery lumen; however, much of the information about the coronary artery wall and plaque composition cannot be captured using this technique [1,2]. Recently, new cardiac imaging techniques such as intravascular ultrasound (IVUS), which can be used to depict coronary arteries and assess atherosclerotic plaques, and cardiac computed tomography (CT), which can be used to estimate the degree of coronary artery calcification, have become available. IVUS, because of its ability to better characterize atherosclerotic plaques, promises to become a powerful complementary tool to coronary angiography. In addition, cardiac CT, which can be used to reveal stenotic coronary vessels and study bypass grafts, may be used as a non-invasive method [2].

We report a case of a 62-year-old man in which the diagnosis of a critical stenotic lesion was reached only after performing the three imaging techniques mentioned above (coronary angiography, cardiac CT and IVUS).

## Case presentation

**Figure 1:**
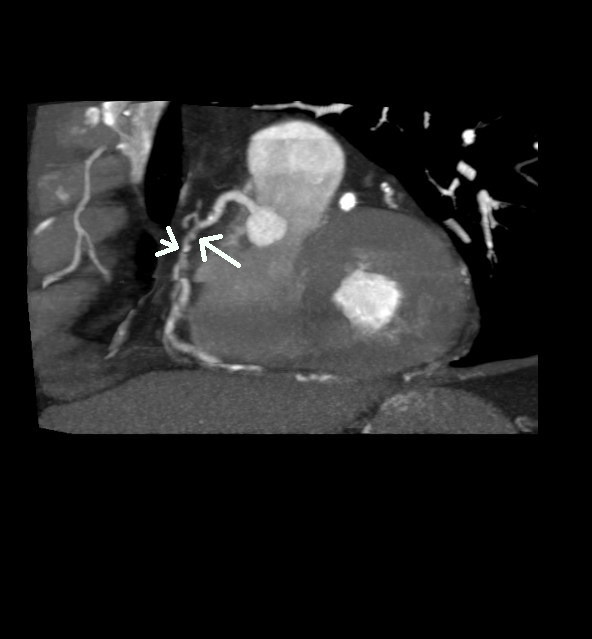
**Stenosis of the right coronary artery (arrow).** Contrast-enhanced slice CT shows the second segment of the right coronary artery in a multiplanar reconstruction. At the site of lumen reduction, a coronary atherosclerotic plaque with positive remodelling can be seen (large arrow). A small calcification is present toward the distal border of the plaque (small arrow).

**Figure 2:**
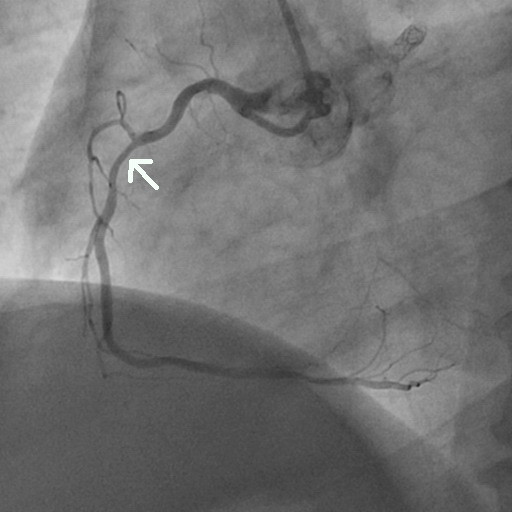
Coronary angiography of the second segment of the right coronary artery showing only mild concentric lumen reduction (arrow).

**Figure 3:**
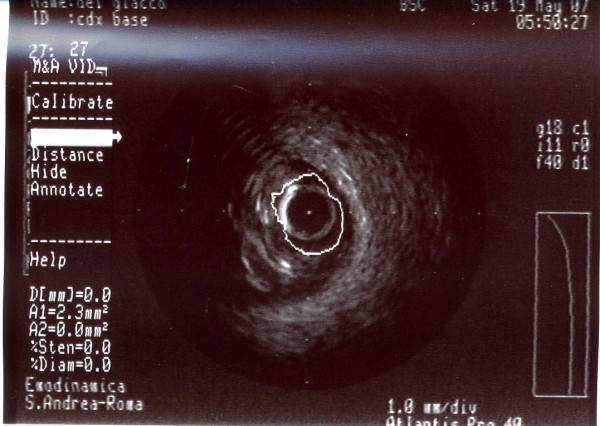
**A-dimensional display mode of IVUS.** The cross-sectional image shows a severe stenosis in the second segment of the right coronary artery (the residual lumen appears as an anechoic area).

The clinical history of the patient, a male smoker who was both hypertensive and hyperlipidemic, began in 2004 when he underwent coronary angiography for unstable angina; this technique established the presence of severe stenosis (80%) of the middle left anterior descending coronary artery (LAD) which was treated with direct stenting with bare metal stent [BMS (Guidant Multi-link Zeta 3.5/18 mm)], and total chronic occlusion of circumflex coronary artery (unsuccessful recanalisation), and atherosclerosis of the middle right coronary artery. In 2005, because of the presence of inducible ischemia on myocardial scintigraphy, the patient underwent a second coronary angiography that showed intrastent restenosis of LAD and was treated with drug eluting stent [DES (Cordis Cypher 3.5/28 mm)]. The patient remained asymptomatic until June 2007 when he was admitted to our hospital after experiencing effort angina; a treadmill test (125 W) revealed signs of ischemia with ST depression V3–V6 and chest pain. After this test, he underwent cardiac CT which documented severe stenosis of the middle right coronary artery (Figure 1). Thus, we decided to perform a therapeutic coronary angiography which, surprisingly, documented only a moderate stenosis (50%) of the right coronary artery (Figure 2). Consequently, following anticoagulation with intravenous heparin (5000 units) and maximal dilation of the coronary artery to prevent spasm with intracoronary nitroglycerin (400 μg), we carried out IVUS that further confirmed the cardiac CT finding. The IVUS study was performed with an automatic pullback (0.5 mm/s) placed distal to the stenotic area near the center of the vessel; the length of the plaque [(16 mm length lesion, lumen area stenosis (LAS) of 70%)] was obtained by counting the number of seconds multiplied by the pullback speed. Minimal lumen area (MLA) was < 4.0 mm^2 ^(exactly 2.7 mm^2^), and plaque burden was >60%. Vessel wall morphology and plaque component were also observed; no bright, echogenic calcium deposits or echolucent structures were recognized in the plaque, whereas a homogeneous, echodense visualization indicated the presence of a fibrous plaque (Figure 3).

**Figure 4:**
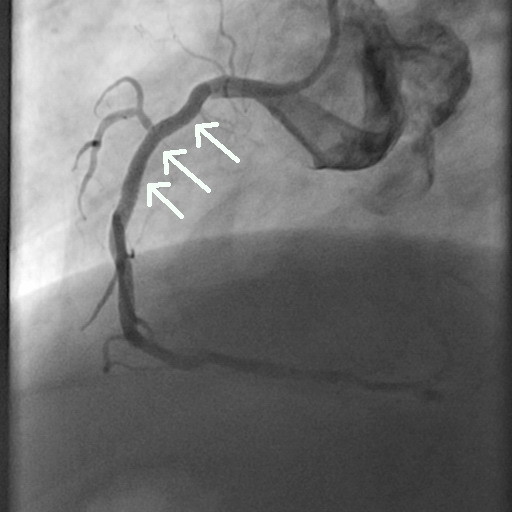
Coronary angiography image obtained just after stent placement in the right coronary artery showing the good revascularization of the artery.

**Figure 5:**
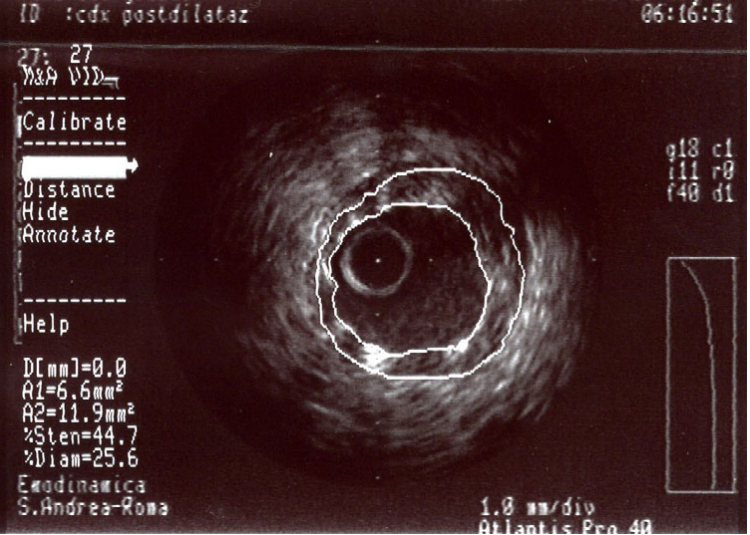
**A-dimensional display mode of IVUS. **The cross-sectional image of the second segment of the right coronary artery obtained after angioplasty shows good deployment of the stent struct to the vessel wall.

After pre-loading with 600 mg of clopidogrel, we decided to treat with angioplasty and direct stenting in the second segment of the right coronary with BMS (Boston Scientific, Libertè 4.0/20 mm) followed by implantation, in overlapping, of another proximal BMS (Boston Scientific, Libertè 4.0/8 mm). Post-dilation with a 4.0/8 mm balloon (Boston Scientific Quantum Maverick) inflated to 18 atm was then performed (Figure 4). At the end of the intervention, a good apposition of the stent struts to the vessel wall with MLA 6.6 mm^2^, LAS <18% and plaque burden 42% was observed in the IVUS study (Figure 5).

**Figure 6:**
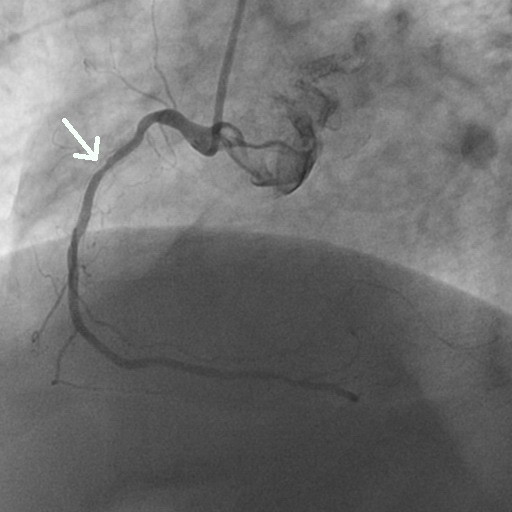
Coronary angiography image (60° left anterior oblique angle) obtained at the six-month follow-up shows 70% diameter stenosis in the second segment of the right coronary artery (arrow).

**Figure 7:**
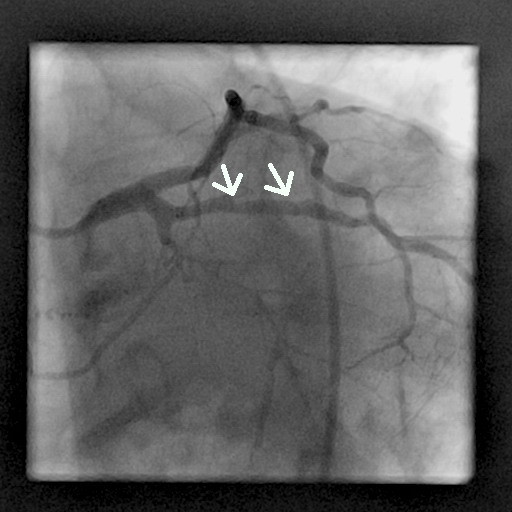
Coronary angiography image (30° right anterior oblique angle) obtained at the six-month follow-up shows 70% diameter stenosis in the second segment of the obtuse marginal branch (arrow).

The patient was discharged on aspirin (100 mg), clopidogrel (75 mg) for one month, losartan (100 mg), athenolol (100 mg), and simvastatin (20 mg), but at the six-month follow-up, he showed new evidence of inducible ischemia on a treadmill test (125 W) (ST depression D2, D3, aVf, V3–V6); subsequently, he underwent a new coronary angiography that showed a right coronary focal intrastent restenosis and severe calcifique stenosis of the middle obtuse marginal branch (Figure 6 and 7).

**Figure 8:**
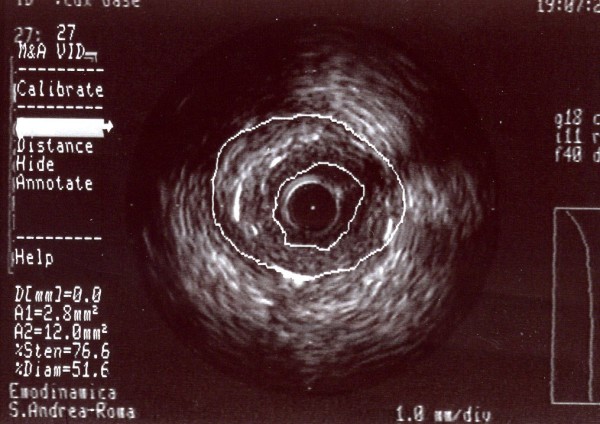
**A-dimensional display mode of IVUS of the same segment as in figure 6. **The cross-sectional intrastent image shows severe stenosis in the second segment of the right coronary artery (the residual lumen appears as an anechoic area). Note a thick fibrous cap consisting of fibrous tissue.

**Figure 9:**
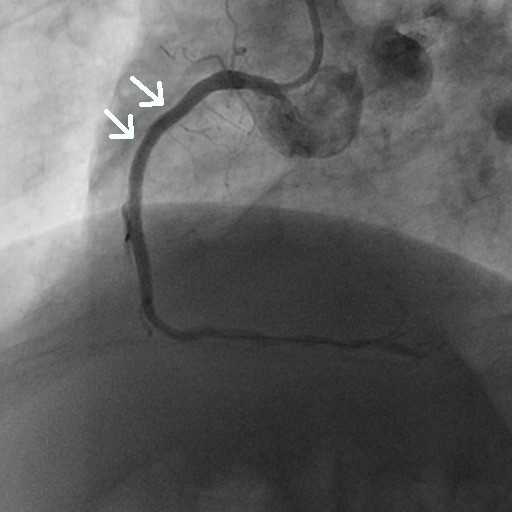
Coronary angiography image obtained just after stent placement in the right coronary artery showing the good revascularization of the artery.

**Figure 10:**
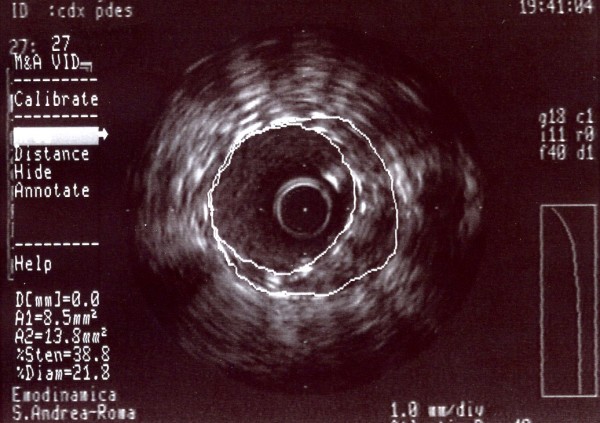
**A-dimensional display mode of IVUS.** The cross-sectional image of the second segment of the right coronary artery obtained after angioplasty shows good deployment of the stent structure to the vessel wall.

We then performed an IVUS study to quantify the stenosis of the right coronary artery (MLA <4 mm^2^, LAS 60.6%, plaque burden 76.6%) and to characterize the lesion (focal fibrous plaque) (Figure 8). Following the standard procedure for performing angioplasty and direct stenting with DES (Boston Scientific, Taxus Libertè 4.0/28 mm, 4.0/16 mm, 4.0/8 mm, overlapping) and post-dilation with a 4.0/12 mm balloon (Boston Scientific Quantum Maverick) inflated to 20 atm (Figure 9), we also performed an IVUS study to assess the adequacy of the deployment of coronary stents (extent of stent apposition and minimum luminal diameter within the stent) in accordance with ACC/AHA recommendations. The IVUS study showed the following results: MLA 8.5 mm^2^, LAS 27%, plaque burden 38% (Figure 10).

**Figure 11:**
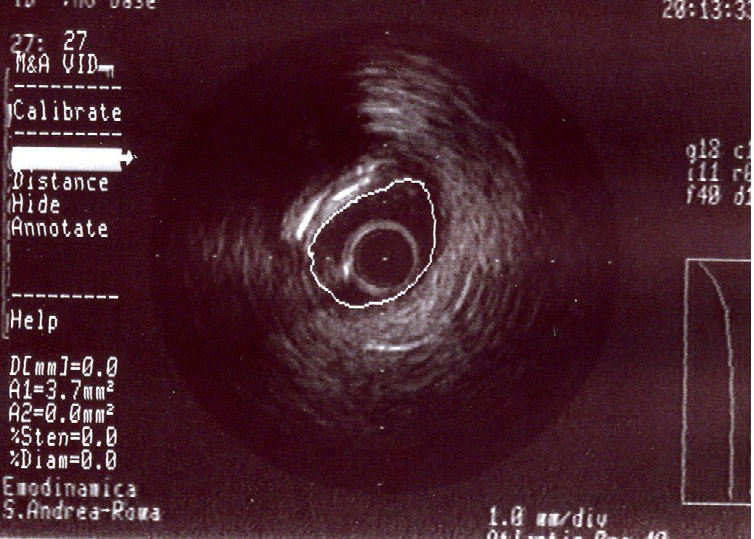
**A-dimensional display mode of IVUS of the same segment as in figure 7.** The cross-sectional image shows severe stenosis in the middle segment of the obtuse marginal branch (the residual lumen appears as an anechoic area). Note the calcified tissue and an ulcerative lesion.

**Figure 12:**
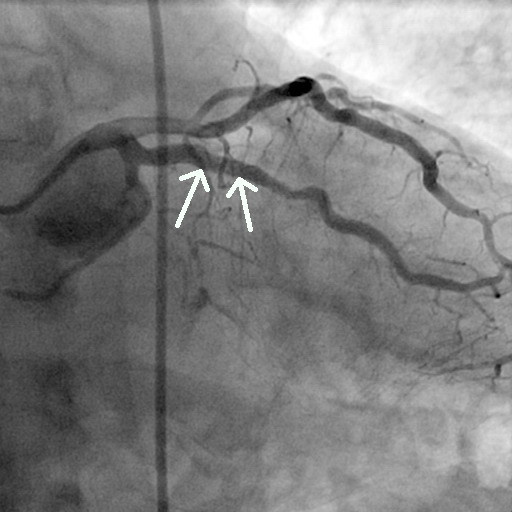
Coronary angiography image obtained just after stent placement in the obtuse marginal branch (arrow) showing good revascularization of the artery.

**Figure 13:**
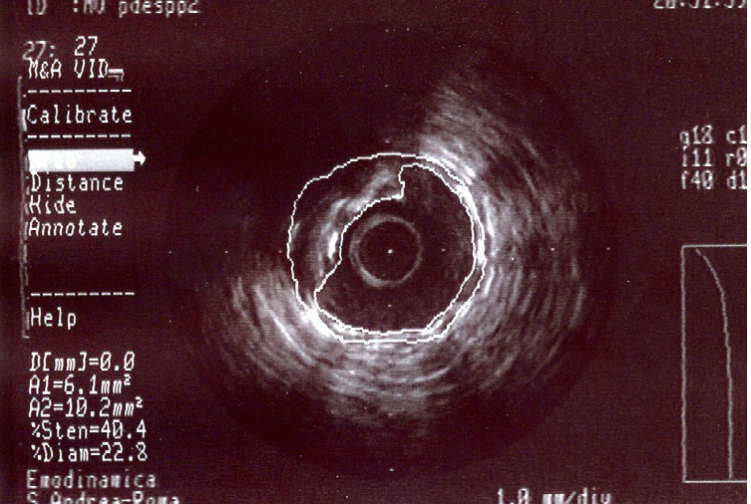
**A-dimensional display mode of IVUS.** The cross-sectional image of the middle segment of the obtuse marginal branch obtained after angioplasty shows good deployment of the stent structure to the vessel wall.

After this first procedure, we also performed an IVUS study to quantify the stenosis of the middle obtuse marginal branch (MLA <4 mm^2^, LAS 52%, plaque burden 61%) and to characterize the lesion (ulcerative and calcifique plaque) (Figure 11). Angioplasty and direct stenting were performed with DES (Cordis, Cypher select 3.5/13 mm, Abbot Xience 3.5/8 mm, overlapping) and post-dilation with a balloon (Boston Scientific Quantum Maverick) 3.5/12 mm until 20 atm (Figure 12). In this coronary artery we also performed an IVUS study that showed the following results: MLA 6.1 mm^2^, LAS 25%, plaque burden 40% (Figure 13).

The patient was discharged on an optimized medical therapy regimen of aspirin (100 mg), atenolol (50 mg b.i.d.), clopidogrel (75 mg) for 12 months and statin (20 mg).

A follow-up scintigraphy 6 months later revealed absence of ischemia.

## Conclusion

This case provides a good illustration of the ineffectiveness of coronary angiography for depicting the wall structure of coronary arteries. In contrast, cardiac CT and IVUS were able to clearly delineate the presence of severe stenosis of the second segment of the right coronary artery. On the other hand, the coronary angiography has been a "battered gold standard" for decades [3]. Recently, cardiac CT has undergone rapid advancements in technological resolution; in fact, the multiple slice spiral version is able to minimize cardiac-motion artifacts, thus allowing for a correct interpretation of the cardiac vascular anatomy and coronary artery disease [4]. According to several studies, the sensitivity and specificity of cardiac CT for revealing the presence of CAD range from 82% to 100% and from 78% to 98%, respectively [4].

IVUS is now a routine procedure in several cardiac catheterization laboratories and is especially used for assessing the severity and morphology of coronary artery lesions and for evaluating indeterminate narrowing of the left main coronary artery or the presence of atherosclerosis progression and/or regression during drug treatment trials [4].

Angiography is always considered to be the gold standard technique for assessing the severity of obstructive luminal narrowing [5], but in some circumstances, it fails to provide a correct interpretation of the lumen vessel stenosis. In fact, some *in vivo *and *post-mortem *human studies have revealed that angiographically normal coronary lesions may have atherosclerosis [3,6-9]. A possible explanation for this is that the presence of diffuse atherosclerosis may induce a remodelling of coronary arteries, thus making it difficult to detect normal reference segments [10]. Furthermore, in the early phases of atherosclerosis, arterial compensatory enlargement may mask the presence of a plaque in the coronary artery lumen [11].

Consequently, other imaging techniques for diagnosing CAD may be necessary.

We aim to emphasize the role that IVUS plays in providing an excellent quantitative measurement of the atherosclerotic plaque and important qualitative information on its composition. Cardiac CT may also give an overall view of coronary atherosclerosis [12]. However, some issues limit the use of IVUS for routine evaluation such as its invasiveness, the related increased risk, the additional time required, and high costs. Although cardiac CT is non-invasive, it cannot be used in patients with significant respiratory or renal failure or a major allergy to contrast material; in addition, cardiac CT is a technique that requires considerable training and is still operator-dependent [12].

In our case, although coronary angiography showed moderate stenosis of the second segment of the right coronary artery, the effort angina experienced by our patient and the presence of a severe stenosis on cardiac CT led us to carry out an IVUS study that allowed us to establish the correct diagnosis.

Unfortunately, our patient from 2004 to present had a total radiation exposure estimated near 120 milliSievert (mSv), a dose that several authors [13-16] consider to raise the cancer risk; according to the conclusions of BEIR VII Phase 2, the linear no-threshold relationship should be used for assessing the detrimental effects of these doses [16]. However, based on the fact that incidence of cancer varies widely depending on age and sex [13] (our patient was an adult male) and that the potential benefit to our patient was greater than the long term risk of cancer, we decided to perform the cardiovascular interventional procedures. Because of its good sensitivity in detecting atherosclerosis in angiographically normal reference segments, IVUS may be a valid and helpful method for completing the diagnosis.

In our case, IVUS has also been very useful for assessing the extent of stent deployment and for determining the minimum luminal diameter within the stent. However, IVUS is an invasive procedure with the potential risks of vessel damage and acute thrombogenic vessel occlusion and should only be used for selected cases.

Therefore, we believe, in accordance with other authors [2], that IVUS is useful in the catheterization laboratory when angiography alone cannot clarify the coronary anatomy.

## List of abbreviations

BMS: bare metal stent; CAD: coronary artery disease; CT: computed tomography; DES: drug eluting stent; IVUS: intravascular ultrasound; LAD: left anterior descending coronary artery; LAS: lumen area stenosis; MLA: minimal lumen area; PTCA: percutaneous transluminal coronary angioplasty.

## Consent

Written informed consent was obtained from the patient for publication of this case report and any accompanying images. A copy of the written consent is available for review by the Editor-in-Chief of this journal

## Competing interests

The authors declare that they have no competing interests.

## Authors' contributions

DMZ performed the coronary angiography and PTCA, participated in the design of the study and in drafting the manuscript. EMZ performed the bibliographic research, participated in the design of the study, drafted the manuscript and prepared the figures. AB participated in the coordination of the manuscript. CN participated in performing the IVUS study. BAP participated in the design of the study. SS participated in the design of the study and prepared the figures. MV participated in the design and coordination of the manuscript.
